# Alternative adenosine Receptor activation: The netrin-Adora2b link

**DOI:** 10.3389/fphar.2022.944994

**Published:** 2022-07-15

**Authors:** Xiaoyi Yuan, Tingting Mills, Marie-Francoise Doursout, Scott E. Evans, Marcos F. Vidal Melo, Holger K. Eltzschig

**Affiliations:** ^1^ Department of Anesthesiology, McGovern Medical School, The University of Texas Health Science Center at Houston, Houston, TX, United States; ^2^ Department of Biochemistry and Molecular Biology, The University of Texas Health Science Center at Houston, Houston, TX, United States; ^3^ Department of Pulmonology, MD Anderson Cancer Center, Houston, TX, United States; ^4^ Department of Anesthesiology, Columbia University, New York, NY, United States

**Keywords:** adenosine, netrin-1, Adora2b, hypoxia, inflammation

## Abstract

During hypoxia or inflammation, extracellular adenosine levels are elevated. Studies using pharmacologic approaches or genetic animal models pertinent to extracellular adenosine signaling implicate this pathway in attenuating hypoxia-associated inflammation. There are four distinct adenosine receptors. Of these, it is not surprising that the Adora2b adenosine receptor functions as an endogenous feedback loop to control hypoxia-associated inflammation. First, Adora2b activation requires higher adenosine concentrations compared to other adenosine receptors, similar to those achieved during hypoxic inflammation. Second, Adora2b is transcriptionally induced during hypoxia or inflammation by hypoxia-inducible transcription factor HIF1A. Studies seeking an alternative adenosine receptor activation mechanism have linked netrin-1 with Adora2b. Netrin-1 was originally discovered as a neuronal guidance molecule but also functions as an immune-modulatory signaling molecule. Similar to Adora2b, netrin-1 is induced by HIF1A, and has been shown to enhance Adora2b signaling. Studies of acute respiratory distress syndrome (ARDS), intestinal inflammation, myocardial or hepatic ischemia and reperfusion implicate the netrin-Adora2b link in tissue protection. In this review, we will discuss the potential molecular linkage between netrin-1 and Adora2b, and explore studies demonstrating interactions between netrin-1 and Adora2b in attenuating tissue inflammation.

## Introduction

Hypoxia and inflammation are highly interdependent (Taylor, 2008; Taylor and Colgan, 2017). Inflammatory lesions are characterized by an imbalance in metabolic supply and demand. The active inflammatory process consumes a large amount of oxygen. For example, polymorphonuclear neutrophils (PMNs) consume such vast amounts of oxygen when activated that they can cause hypoxic imprinting on neighboring stromal or epithelial cells (Campbell et al., 2014). Other inflammatory cells such as natural killer cells (Victorino et al., 2015; Hoegl et al., 2016), eosinophils (Patel et al., 2014; Masterson et al., 2015; Wang et al., 2021a), macrophages (Gao et al., 2020), or T-cells (Sun et al., 2010; Clambey et al., 2012; Ehrentraut et al., 2012; Ehrentraut et al., 2013; Yuan et al., 2019) contribute to shaping the metabolic environment in inflammatory or infectious foci (Koeppen et al., 2011a; Gumbert et al., 2020). At the same time, the supply of metabolites from the bloodstream is often diminished due to microvascular occlusions, edema or shunting (Eltzschig and Collard, 2004). In addition, alterations of specific metabolites (e.g., accumulation of succinate) can further shape a hypoxic microenvironment and contribute to transcriptional reprogramming (Haeberle et al., 2008; Eckle et al., 2013a; Vohwinkel et al., 2021). Many studies have found that during hypoxia-induced inflammation (inflammatory hypoxia), extracellular levels of adenosine are elevated (Ohta and Sitkovsky, 2001; Sitkovsky et al., 2004; Sitkovsky and Lukashev, 2005; Thiel et al., 2005), and implicate extracellular adenosine signaling in an endogenous feedback loop to attenuate hypoxia-induced inflammation (Cronstein, 1994; Eltzschig and Carmeliet, 2011; Eltzschig et al., 2012).

Adenosine is part of a group of biomolecules termed purines, defined as heterocyclic aromatic molecules (Eltzschig, 2013). These molecules belong to the most ancient and conserved biochemical molecules during evolution (Miller and Urey, 1959). These relatively simple biochemical molecules are fitted together from adenine and guanine, resembling the most critical building block for mammalian genes (Fredholm and Verkhratsky, 2010). Therefore, the purine adenosine has earned its place as the biomolecular building block of the genetic code and as part of the universal biological energy currency, adenosine triphosphate (ATP) (Khakh and Burnstock, 2009; Eltzschig, 2013). However, adenosine plays diverse roles in phyeiological and pathophysiological conditions (Khakh and Burnstock, 2009). Beyond these functions, adenosine has been recognized as a signaling molecule through the activation of four receptors named A1, A2A, A2B, and A3 receptors (Adora1, Adora2a, Adora2b, Adora3). These G-protein coupled receptors have many biological functions. For example, activation of Adora1 slows the heart rate, allowing adenosine injection to be used for treating supraventricular tachycardia (Koeppen et al., 2009). Adora2a is expressed on immune cells, such as PMNs (Cronstein et al., 1990) and T-cells (Yang et al., 2006a), and has been shown to dampen harmful inflammation (Ohta and Sitkovsky, 2001; Hasko and Cronstein, 2004). Adora3 has been implicated in mast cell activation and the pathogenesis of asthmatic airway disease (Jin et al., 1997; Zhong et al., 2003).

In contrast to the other three adenosine receptors, the Adora2b is somewhat unique in its role for hypoxia adaptation (Eltzschig et al., 2003; Kong et al., 2006) and has been considered a safety signal during inflammatory hypoxia (Grenz et al., 2011a; Koeppen et al., 2011b). Two features make Adora2b well suited to hypoxia adaptation. First, Adora2b is transcriptionally induced by hypoxia-inducible transcription factor HIF1A (Kong et al., 2006; Poth et al., 2013), and therefore levels of Adora2b are highest during hypoxia or inflammatory states (Eckle et al., 2013b; Eckle et al., 2014; Hoegl et al., 2015; Wang et al., 2021b). Second, it is the most “insensitive” of the four adenosine receptors, requiring the highest adenosine concentrations to be activated (Feoktistov and Biaggioni, 1997; Aherne et al., 2011; Koeppen et al., 2011b). Such high concentrations of extracellular adenosine are present during hypoxia and inflammation and activate the Adora2b receptor (Feoktistov and Biaggioni, 1997; Feoktistov and Biaggioni, 2011).

Interestingly, several studies have suggested an alternative adenosine independent mechanism of Adora2b activation, particularly during hypoxia or inflammation. This process involves the neuronal guidance molecule netrin-1. Netrin-1 is one of neuronal guidance molecules that are critical for neuronal development by either attracting or repelling developing neurons (Serafini et al., 1994; Serafini et al., 1996; Corset et al., 2000; Mirakaj and Rosenberger, 2017; Keller et al., 2021). Several studies suggest that during inflammatory conditions, netrin-1-elicited organ protection during inflammatory hypoxia is dependent on Adora2b signaling (Rosenberger et al., 2009). Additionally, other studies indicate that netrin-1 is a direct ligand of Adora2b (Stein et al., 2001; Feoktistov and Biaggioni, 2011). In the present review, we will first discuss the role of Adora2b during hypoxia and inflammation. Subsequently, we will explore studies linking netrin-1 with Adora2b signaling during hypoxia, inflammation or ischemia and reperfusion. Finally, we will discuss potential molecular mechanisms connecting netrin-1 with Adora2b and the evidence that argues for and against a direct activation of the Adoar2b by netrin-1.

## Extracellular adenosine signaling during hypoxia and inflammation

That hypoxia is associated with increased extracellular adenosine levels has been known for many years. For example, studies from the early 1990s showed that plasma adenosine levels in rats rose from approximately 80 nM at baseline to approximately 190 nM following exposure to ambient hypoxia (8% oxygen) (Phillis et al., 1992). Hypoxia-driven increases of extracellular adenosine have been implicated as an endogenous feedback mechanism to promote hypoxia adaptation. For example, recent reports indicate that adenosine levels in plasma are induced in a rapid manner after high altitude exposure. Importantly, the induction is amplified upon re-ascent. This observation has been subsequently linked with faster adaptation to high altitudes and more rapid acclimatization (Liu et al., 2016; Song et al., 2017; Sun et al., 2017).

**FIGURE 1:**
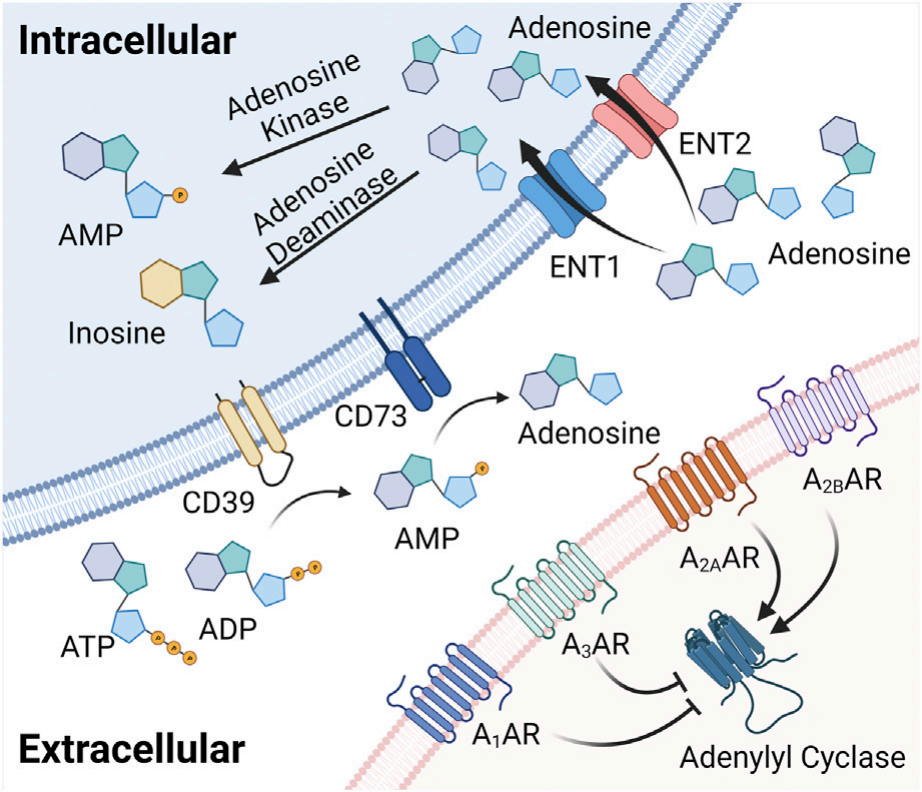
Extracellular adenosine generation, signaling, and termination. Extracellular adenosine can be enzymatically produced from precursor nucleotides, which involves a two-step enzymatic process involving the conversion from ATP or ADP to AMP. This conversion is controlled by ecto-apyrase CD39. The second step for the extracellular generation of adenosine is mediated by the ecto-5′-nucleotidase CD73, a glycosylphosphatidylinositol (GPI)-anchored protein that converts AMP to adenosine, and functions as an extracellular pacemaker for the production of adenosine. Once liberated into the extracellular compartment, adenosine binds to four G-protein coupled adenosine receptors, including the Adora1, Adora2a, Adora2b and the Adora3 adenosine receptor with specific biologic functions during health or disease. Finally, extracellular adenosine signaling is terminated *via* ENTs to be uptaken into the intracellular space and converted to inosine or AMP.

Studies in genetic and pharmacologic models with altered adenosine production have provided insight into mechanisms controlling extracellular adenosine levels during hypoxia and inflammation. Extracellular adenosine can be enzymatically produced from precursor nucleotides, which involves a two-step enzymatic process (Figure 1). The first step involves the conversion of precursor nucleotides–such as ATP or ADP to AMP (Garcia-Morales et al., 2016a; Bowser et al., 2017a; Bowser et al., 2017b; Bowser et al., 2018). This conversion is controlled by ecto-apyrase CD39 (Kaczmarek et al., 1996; Enjyoji et al., 1999; Robson et al., 2001; Robson et al., 2005). During injurious conditions such as hypoxia or inflammation, many cells release precursor nucleotides (Eltzschig et al., 2006a; Faigle et al., 2008; Colgan and Eltzschig, 2012), and therefore the extracellular production of AMP is dramatically increased (Eltzschig et al., 2006b; Eckle et al., 2007a; Kohler et al., 2007; Eltzschig et al., 2009a; Friedman et al., 2009; Reutershan et al., 2009; Hart et al., 2010; Le et al., 2019). The second step for the extracellular generation of adenosine is mediated by the ecto-5′-nucleotidase CD73, a glycosylphosphatidylinositol (GPI)-anchored protein that converts AMP to adenosine, and functions as extracellular pacemaker for the production of adenosine (Hansen et al., 1995; Weissmuller et al., 2005; Eltzschig et al., 2008; Zhang et al., 2013). Studies in mice with genetic deletion of *Cd39* or *Cd73* suggested that these animals have more severe vascular leakage and inflammatory responses during hypoxia exposure (Eltzschig et al., 2003; Eltzschig et al., 2004; Thompson et al., 2004). Similarly, they experience more profound tissue injury and inflammation when exposed to models of acute respiratory distress syndrome (ARDS) (Eckle et al., 2007a; Eckle et al., 2008a; Reutershan et al., 2009; Koeppen et al., 2011c), myocardial ischemia and reperfusion (Eckle et al., 2006; Eckle et al., 2007b; Kohler et al., 2007; Eckle et al., 2011), liver injury (Hart et al., 2008a; Hart et al., 2008b; Hart et al., 2010), or intestinal inflammation (Hart et al., 2008c; Hart et al., 2008d; Louis et al., 2008; Friedman et al., 2009; Hart et al., 2009). Taken together, these studies indicate that during inflammatory hypoxia, the production of extracellular adenosine is elevated and serves as an endogenous feedback signal to diminish excessive inflammation (Eltzschig et al., 2012).

**FIGURE 2:**
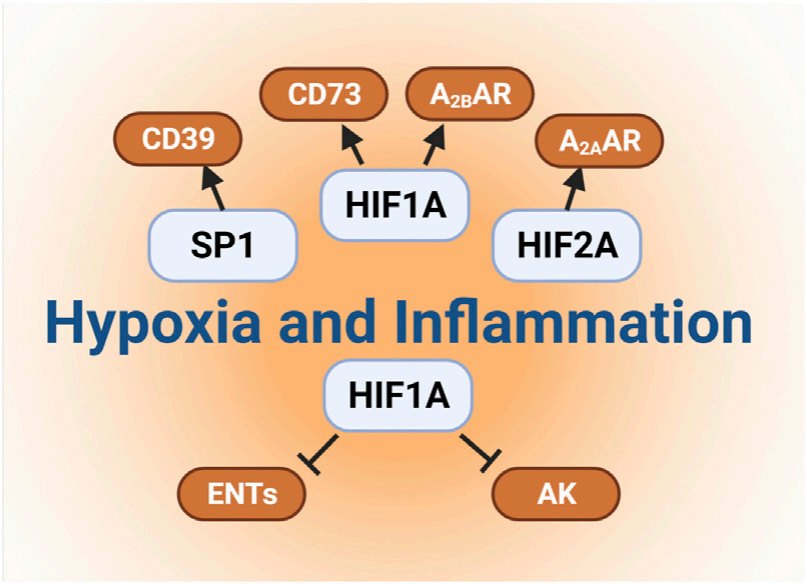
Links between hypoxia and extracellular adenosine. Several studies have elucidated the molecular mechanisms involved in hypoxia-dependent enhancement of extracellular adenosine signaling. Firstly, hypoxia and concomitant HIF1A stabilization directly induces the expressional levels of CD73. Secondly, hypoxia dependent CD39 induction is controlled by a transcriptional mechanism involving SP1 Furthermore, HIF1A and HIF2A stabilization results in the enhancement of adenosine receptors expression, such as Adora2b and Adora2a, respectively. Finally, previous studies also implicate HIF1A in the repression of both ENTs and adenosine kinases, which indirectly promotes the additional elevations of extracellular signaling events.

Several studies have elucidated the molecular mechanisms involved in hypoxia-dependent enhancement of extracellular adenosine signaling (Figure 2). These studies identified a central regulatory role of HIF1A by regulating the expressional levels of CD73. Hypoxia signaling through HIFs resembles an adaptive signaling pathway that has been selected from ancient atmospheres for survival benefit under flunctuating oxygen levels (Taylor and McElwain, 2010). During hypoxia or inflammation, HIF1A is stabilized and forms a transcriptionally active heterodimer with HIF1B. Subsequent translocation to the nucleus and binding to hypoxia response elements (HREs) in hypoxia-controlled target genes typically causes induction of the gene product (Eltzschig et al., 2014; Ju et al., 2016; Semenza, 2020). Notable HIF target genes include, for example, erythropoietin, the group of enzymes controlling the glycolytic flux of carbohydrate intermediates, and vascular endothelial growth factor (Wang and Semenza, 1993; Semenza et al., 1994; Wang et al., 1995; Forsythe et al., 1996; Semenza et al., 1996). The discovery of the HIF pathway was recognized by the 2019 Nobel Prize in medicine or physiology (Fandrey et al., 2019; Colgan et al., 2020). While most commonly HIF binding causes induction of target genes (Zheng et al., 2009), there are also many instances where HIF1A activity causes gene repression (Eltzschig et al., 2005; Loffler et al., 2007; Morote-Garcia et al., 2008; Morote-Garcia et al., 2009; Ju et al., 2021), such as by the induction of HIF-dependent microRNAs (Ferrari et al., 2016a; Ferrari et al., 2016b; Neudecker et al., 2016; Neudecker et al., 2017a; Lee et al., 2020a). Studies of hypoxia exposure of epithelial cells or vascular endothelia demonstrate that both CD39 and CD73 are induced by hypoxia (Synnestvedt et al., 2002; Eltzschig et al., 2003). While CD39 is controlled by SP1 (Eltzschig et al., 2009a; Hart et al., 2010), the promoter of CD73 contains an HRE, and studies with transcription factor binding and promoter constructs directly implicate HIF1A in the transcriptional induction of CD73 (Synnestvedt et al., 2002; Garcia-Morales et al., 2016b). Additional molecular mechanisms of HIF-dependent increases in extracellular adenosine during hypoxia and inflammation include transcriptional repression of adenosine uptake and metabolism (Eltzschig et al., 2005; Loffler et al., 2007; Morote-Garcia et al., 2008). Adenosine signaling is terminated by equilibrative nucleoside transporters, ENT1 or ENT2 mediated uptake of extracellular adenosine towards intracellular spaces (Rose et al., 2011; Eckle et al., 2013b; Morote-Garcia et al., 2013; Aherne et al., 2018; Wang et al., 2021b) (Figure 1). Previous studies implicate HIF1A in the repression of both ENT1 (Eltzschig et al., 2005) and ENT2 (Morote-Garcia et al., 2009) during hypoxia or inflammatory diseases, leading to additional elevations of extracellular signaling events (Loffler et al., 2007). Similarly, HIF1A causes transcriptional repression of the adenosine kinase (Morote-Garcia et al., 2008), a response that dampens intracellular adenosine metabolism from adenosine to AMP, thereby functioning to further enhance adenosine signaling events in the extracellular compartment (Decking et al., 1997; Ferrari et al., 2016a; Ferrari et al., 2016b; Ferrari et al., 2016c; Yuan et al., 2021). Taken together, these studies accentuate the functional role of HIF1A in the control of extracellular adenosine signaling during hypoxia and inflammation (Poth et al., 2013).

## Adora2b during hypoxia and inflammation

Once liberated into the extracellular compartment, adenosine binds four G-protein coupled adenosine receptors: Adora1, Adora2a, Adora2b and Adora3 (Figure 1) (Hasko and Cronstein, 2004; Sitkovsky et al., 2004; Sitkovsky and Lukashev, 2005; Hasko et al., 2008; Eltzschig, 2009; Idzko et al., 2014a; Idzko et al., 2014b). Each of these receptors has been associated with specific biologic functions during health or disease states (Chen et al., 2013). For example, the Adora1 was suggested to mediate the heart-rate slowing effects of adenosine (Koeppen et al., 2009). As such, functional studies implicate this receptor in cardio-protection during ischemic pre- or post-conditioning (Matherne et al., 1997; Reichelt et al., 2005), an experimental strategy where short periods of non-lethal ischemia can be applied to increase myocardial resistance to ischemia (Eckle et al., 2006; Redel et al., 2008). The Adora2a is expressed on inflammatory cells and has been shown to dampen acute inflammatory responses in a variety of models (Cronstein et al., 1990; Ohta and Sitkovsky, 2001; Sitkovsky et al., 2004; Thiel et al., 2005; Hasko et al., 2008). The Adora3 has been shown to be expressed on mast-cells and has been implicated in asthmatic airway disease (Salvatore et al., 2000; Zhong et al., 2003), while studies in *Adora3* ^*−/−*^ mice show elevated blood pressure, aggressiveness and hypoalgesia (Ledent et al., 1997). The Adora2b receptor has been identified as a “low-affinity” receptor (Feoktistov and Biaggioni, 1997; Feoktistov and Biaggioni, 2011), and since it appears to have many similar functions to the Adora2a (e.g., both receptors promote intracellular cAMP levels), it was initially thought to be redundant or lesser physiologic compared to the other adenosine receptors (Aherne et al., 2011). However, several factors have led to a rethinking of Adora2b, and have identified the Adora2b as a critical adenosine receptor during adaptation to hypoxia or inflammation. First, the fact that signaling events through the Adora2b require higher adenosine levels than other three adenosine receptors highlights that the Adora2b is particularly active during states of elevated adenosine levels, such as during hypoxia and inflammation (Van Linden and Eltzschig, 2007; Eltzschig et al., 2009b; Koeppen et al., 2011b; Wen et al., 2011; Zhang et al., 2011; Grenz et al., 2012; Karmouty-Quintana et al., 2012). Secondly, several studies provide evidence for selective induction of the Adora2b during hypoxia or inflammation. For example, a screen for transcriptional responses in human vascular endothelial cells exposed to hypoxia (2% oxygen) revealed that only the Adora2b transcript levels were induced (Eltzschig et al., 2003). Functional studies in *Adora2b* ^*−/−*^ mice demonstrate that these mice experience more profound vascular inflammation, including significantly increased leukocyte adhesion to the vasculature and increased pro-inflammatory cytokine levels upon stimulation with LPS (Yang et al., 2006b). Similarly, *Adora2b* ^*−/−*^ mice are more prone to obesity, delayed glucose clearance and augmented insulin levels compared to controls (Johnston-Cox et al., 2012). Other studies highlight that *Adora2b* ^*−/−*^ mice are susceptible to developing vascular lesions in vascular injury models (Yang et al., 2008). Together with other studies (Eltzschig et al., 2003; Eltzschig et al., 2004), these findings highlight functional roles for the Adora2b during tissue inflammation and hypoxia, as an endogenous feedback cue to control excessive inflammation.

## Discovery and function of netrin-1 as a neuronal guidance molecule

Netrin-1 was initially discovered as a neuronal guidance molecule (Serafini et al., 1994; Serafini et al., 1996). The name netrin comes from the Sanskrit word “netr,” meaning “one who guides”. Netrin-1 belongs to the family of netrins, which are composed of secreted proteins that are critical to the developing brain, due to their function to attract or repel growing axons. Neuronal guidance is a critical aspect of brain development, where neurons send out axons to reach their correct targets (Colamarino and Tessier-Lavigne, 1995). Purification of proteins derived from embryonic chick brain has led to the identification of the protein netrin-1, which showed commissural axon outgrowth-promoting activity (Serafini et al., 1994). In addition, studies in gene-targeted mice for netrin-1 (*Ntn1* ^*−/−*^mice) revealed impaired spinal commissural axon projections, implicating functional roles of netrin-1 in axon guidance (Serafini et al., 1996). The neurologic defects in homozygous *Ntn1* ^*−/−*^ mice are so severe that these mice are born and die withing a few days due to significant neurologic defects including the lacking of suckling, and inability to move their forelimbs (Serafini et al., 1996). Subsequent studies used transgenic mice with a “floxed” netrin-1 gene, so that studies in adult mice would be possible (Brunet et al., 2014; Varadarajan et al., 2017; Zhu et al., 2019; Li et al., 2021). Nevertheless, the above described early studies in mice gene-targeted for netrin-1 established netrin-1 as a guidance molecule that functions during vertebral brain development (Serafini et al., 1996).

## Netrin-1 as a guidance cue during inflammation

**FIGURE 3:**
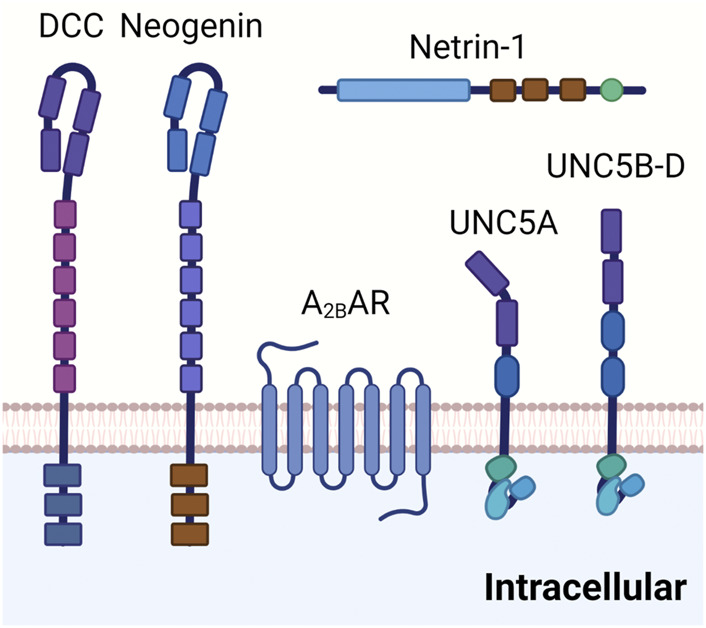
Netrin-1 receptors. DCC is a transmembrane protein composed of four immunoglobulin like repeats and six fibronectin type II like repeats on the extracellular site. It is involved in the netrin-1 mediated bi-functional guidance of neurons. Neogenin shares a similar structure to DCC and has been implicated in tissue morphogenesis, angiogenesis, as well as axon guidance. Adora2b has been identified as a netrin-1 coreceptor in netrin-1 signaling in certain tissues, although detailed mechanisms are still under investigation. UNC5 receptors (UNC5A-D) are composed of two immunoglobulin like repeats and two throbomspondin domains in the extracellular area. UNC5 receptors have been suggested in the long range and short range repulsion during axon guidance.

The properties and functions on netrin-1 within the CNS as neuronal guidance molecule make it an ideal candidate for also guiding inflammatory cell trafficking events. In fact, several other neuronal guidance molecules (Konig et al., 2012; Mirakaj et al., 2012; Kohler et al., 2013; Kohler et al., 2020) have been implicated in immunomodulation and coordination of inflammatory events or resolution of inflammation (Mirakaj and Rosenberger, 2017; Keller et al., 2021). The unique characteristic of netrin-1 to repulse or abolish the attraction of developing neuronal cells *via* signaling events through the UNC5b receptor makes it a perfect candidate gene for coordinating inflammatory cell migration (Figure 3). In line with this hypothesis, studies showed that netrin-1 is expressed on vascular endothelial cells, where its expression can be regulated by infection or inflammation. Similarly, UNC5b was found to be expressed on leukocytes and interacts with netrin-1 as migration inhibitor to different chemotaxis (Ly et al., 2005).

Subsequent studies suggest that netrin-1 can also function to promote atherosclerosis by entrapping macrophages in plaques (van Gils et al., 2012). In line with these findings, a recent study demonstrates that silencing of netrin-1 in the myeloid lineage promotes the resolution of inflammation and plaque regression (Schlegel et al., 2021). Netrin-1 signaling can also function as a macrophage retention signal for the promotion of chronic inflammation and insulin resistance in adipose tissue (Ramkhelawon et al., 2014) or in the pathogenesis of abdominal aortic aneurism in vascular smooth muscle cells (Hadi et al., 2018). Other studies suggest a functional role of netrin-1 signaling in osteoclast differentiation (Mediero et al., 2015), inflammatory arthritis (Mediero et al., 2016; Zhu et al., 2019), or pulmonary fibrosis (Gao et al., 2021). In line with the concept that netrin-1 can have highly diverse signaling functions, a very elegant study implicated netrin-1 signaling in the resolution process of inflammation, an intricate process involving pro-resolving mediators (Serhan and Levy, 2018; Mirakaj, 2021). During zymosan-initiated peritoneal inflammation, the vagus nerve was found to regulate the local expression of netrin-1 (Mirakaj et al., 2014). A vagotomy results in delayed resolution through inhibition of pro-resolving mediators. Using genetic studies, the authors found that in mice with partial netrin-1 deficiency, pro-resolving mediator resolvin D1 failed to reduce neutrophil influx, thus dampening the resolution of peritonitis compared with controls. Similarly, when human monocytes were treated with recombinant netrin-1, their production of lipid proresolving mediators was increased. These findings suggest that the vagus nerve controls both netrin-1 and pro-resolving programs (Mirakaj et al., 2014). Beyond those studies, and pertinent to the present review, several studies over the past 2 decades have repeatedly shown that the anti-inflammatory and pro-resolution role of netrin-1 signaling can be mediated through the Adora2b adenosine receptor (Eltzschig, 2009; Aherne et al., 2013).

## Linkages between Adora2b and netrin-1 signaling

Not long after its original discovery as a neuronal guidance molecule (Serafini et al., 1994; Colamarino and Tessier-Lavigne, 1995; Serafini et al., 1996), a study reported a previously unrecognized interaction between netrin-1 and the Adora2b adenosine receptor (Corset et al., 2000). This study was based on the notion that the interaction of netrin-1 with its receptor deleted in colorectal carcinoma (DCC) might involve an additional co-receptor, since netrin-1 protein only co-immunoprecipitate with DCC if cross-linked. Moreover, netrin-1 did not bind to a soluble fusion protein of the extracellular domain of DCC directly *in vitro* (Meyerhardt et al., 1999). To find such a co-receptor, a subsequent study utilized a two-hybrid screen of human brain complementary DNA and identified a fragment corresponding to the last 23 amino acids of the intracellular domain of the Adora2b with potential binding to intracellular domain of DCC. Using co-immunoprecipitation, they demonstrated a direct interaction of the Adora2b with DCC, but only in the presence of netrin-1. Additionally, they showed that the Adora2b can serve as a netrin-1 receptor, including the induction of cAMP elevations upon binding of netrin-1 to the Adora2b. Finally, they performed studies on netrin-1 mediated axon growth and described that netrin-1-mediated outgrowth of dorsal spinal cord axons requires Adora2b signaling (Corset et al., 2000). In contrast to those findings, a subsequent study found that netrin-1 directly regulates axon guidance, independent of the Adora2b (Stein et al., 2001). Moreover, another study demonstrated that netrin-1 does not bind to Adora2b. However, Adora2b activation by adenosine analogs facilitates neuron response to netrin-1 by reducing the levels of cell surface Unc-5 netrin receptor A (UNC5A) receptor, thereby supporting an indirect interaction between Adora2b and netrin-1 in the developing brain (McKenna et al., 2008). Recently, the notion that netrin-1 and Adora2b signaling are linked was rejuvenated in a study of hypoxia-associated inflammation, where netrin-1 released from intestinal epithelial cells dampened inflammatory responses by activating Adora2b receptors expressed on polymorphonuclear granulocytes (PMNs) (Rosenberger et al., 2009), a finding that was subsequently confirmed in many other studies (He et al., 2014; Schlegel et al., 2016; Zhou et al., 2019; Chen et al., 2020).

## Coordination of netrin-1 and Adora2b signaling by hypoxia

As discussed above, the interaction of netrin-1 with the Adora2b was first established in brain development (Corset et al., 2000), but did not find consistent support from subsequent studies (Stein et al., 2001; McKenna et al., 2008). However, studies on inflammatory responses explored the possibility of the Adora2b-netrin-1 link (Rosenberger et al., 2009). The first study linking Adora2b signaling with netrin-1 during inflammation was based on the hypothesis that hypoxia would elicit endogenous adaptive responses that could dampen hypoxia-associated inflammation. In line with this hypothesis, the authors found that netrin-1 is expressed in intestinal epithelial cells and is induced by hypoxia. Studies on the mechanism of hypoxia-dependent induction of netrin-1 identified an HRE within the promoter region of netrin-1 that interacts with HIF1A during conditions of hypoxia, as shown by studies of netrin-1 promoter constructs, chromatin immunoprecipitation, and *in vitro* and *in vivo* studies of HIF1A mutations (Rosenberger et al., 2009). For example, wild-type mice would have robust induction of netrin-1 in their intestinal epithelial cells upon exposure to ambient hypoxia (4 h in 8% oxygen), while mice with intestinal epithelial Hif1a deletion would fail to induce netrin-1 expression (Rosenberger et al., 2009; Grenz et al., 2012). Subsequent functional studies demonstrated that netrin-1 signaling dampens hypoxia-associated inflammation *via* signaling events through the Adora2b receptor expressed on PMNs. Several subsequent studies confirmed the role of HIF1A in inducing netrin-1, including studies in macrophages exposed to ambient hypoxia (Ramkhelawon et al., 2013) or during LPS induced inflammation (Berg et al., 2021). In this later study, an unbiased screen revealed that netrin-1 was the highest induced neuronal guidance molecule released from macrophages exposed to LPS. Similar to the above studies, the authors found an important role of HIF1A in inducing netrin-1 upon LPS stimulation, and demonstrated in functional *in vivo* studies, that mice with myeloid deletion of netrin-1 (*Ntn1* ^*loxp/loxp*^ LysMCre + mice) experienced exaggerated mortality and lung inflammation. More detailed examination of the *Ntn1* ^*loxp/loxp*^ LysMCre + mice demonstrated a functional role of netrin-1 in repelling natural killer cells, a response which could potentially implicate Adora2b signaling (Berg et al., 2021). Other studies found upregulation of netrin-1 by hypoxia during cancer (Chen et al., 2016; Jin et al., 2019), or in promoting anti-apoptotic function in endothelial progenitor cells under hypoxia conditions (Jiang et al., 2022). In conjunction with previous studies demonstrated that the Adora2b is a classic HIF target gene (Kong et al., 2006), and is selectively induced during hypoxia (Eltzschig et al., 2003; Schingnitz et al., 2010), ischemia (Eckle et al., 2007b; Eckle et al., 2008b; Grenz et al., 2008) or inflammation (Frick et al., 2009; Hart et al., 2009; Csoka et al., 2010; Ehrentraut et al., 2012; Eckle et al., 2014; Aherne et al., 2015; Hoegl et al., 2015), the above findings introduce the possibility that conditions of hypoxia coordinate Adora2b and netrin-1 signaling. During inflammatory hypoxia, netrin-1 expression is increased, Adora2b is induced, leading to increased Adora2b-dependent signaling events. Therefore, it is not surprising that previous studies of hypoxia-associated inflammation have provided links between netrin-1 and Adora2b signaling (Aherne et al., 2013).

## Netrin-1 in alternative adenosine receptor activation during inflammation or ischemia and reperfusion

As described above, inflammatory hypoxia is associated with heightened expression of netrin-1 and Adora2b receptors, setting the stage for interactions between netrin-1 and Adora2b signaling. Several studies have found a functional role of the netrin-1-Adora2b link during studies that examine inflammatory conditions in tissue compartments where hypoxia-associated inflammation and changes in metabolic supply and demand cause stabilization of HIFs. Examples include ARDS, inflammatory bowel disease, myocardial and hepatic ischemia and reperfusion injury.

### Acute respiratory distress syndrome

**FIGURE 4:**
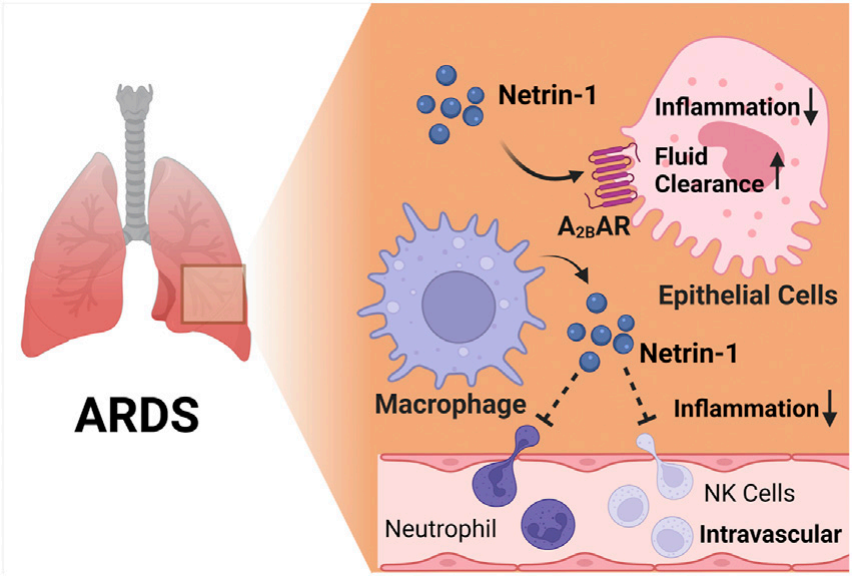
The netrin-1/Adora2b link in acute respiratory distress syndrome (ARDS). Several studies implicate that netrin-1 provide lung protection during ARDS. In alveolar epithelial cells, netrin-1 enhances alveolar fluid clearance and reduces alveolar inflammation, and this process is highly dependent on Adora2b signaling. Furthermore, macrophage derived netrin-1 inhibits neutrophil and natural killer cell recruitment, leading to reduced inflammation during endotoxin induced ARDS.

ARDS is an inflammatory disease of the lungs characterized by acute onset, the presence of bilateral pulmonary edema in the absence of left heart failure, and profound hypoxia with PaO2/FiO2 less than 300 mmHg (Lee et al., 2019; Li et al., 2020). Patients frequently require mechanical ventilation (Williams et al., 2021), and ARDS carries a very high rate of morbidity and mortality (Eckle et al., 2009; Ranieri et al., 2012; Dengler et al., 2013; Bellani et al., 2016). Based on its effect on repelling leukocyte infiltration, one of the first studies to examine functional roles of netrin-1 during ARDS used injurious mechanical ventilation to induce ARDS, as neutrophilia is consistently observed in this model (Eckle et al., 2008a; Koeppen et al., 2011c). Studies of mice with partial netrin-1 deficiency showed increased lung inflammation during injurious mechanical ventilation, and could be resuscitated by treatment with recombinant netrin-1 *via* inhalation or intravenous administration (Mirakaj et al., 2010). Another study confirmed the protective effects of netrin-1 treatment using a large animal model (Mutz et al., 2010). In this study, ARDS was induced by an intravenous infusion of LPS, and mice were subsequently treated with intravenous netrin-1 or inhaled netrin-1. Netrin-1 treatment provided lung protection by reducing inflammatory markers and histologic injury, and computed tomography corroborated attenuated pulmonary damage in both netrin-1 treatment arms (Mutz et al., 2010). Additional studies implicate HIF1A in the induction of netrin-1 and its protection during ARDS, and particularly implicate myeloid-derived netrin-1 in lung protection (Berg et al., 2021). Importantly, several previous studies demonstrate that HIF1A is stabilized during ARDS, and can function to dampen alveolar inflammation (Eckle et al., 2013a; Eckle et al., 2014; Vohwinkel et al., 2015; Garcia-Morales et al., 2016a; Vohwinkel et al., 2021). Studies on the signaling mechanism involved in netrin-1-elicited lung protection indicate that netrin-1 requires Adora2b signaling. For example, the lung protective effects during treatment with recombinant netrin-1 were completely abolished when applied in mice with global deletion of the Adora2b (*Adora2b* ^*−/−*^ mice) (Mirakaj et al., 2010). Moreover, other studies implicate netrin-1 in promoting alveolar fluid clearance by enhancing Adora2b signaling during ARDS (He et al., 2014). These findings were based on previous studies that had demonstrated links between adenosine signaling and fluid clearance during ARDS (Factor et al., 2007; Kreindler and Shapiro, 2007). Indeed, measurements of alveolar fluid clearance directly implicate Adora2b signaling in the enhancement of amiloride-sensitive fluid transport and elevations of pulmonary cAMP during ARDS induced by mechanical ventilation, suggesting that Adora2b agonist treatment (such as BAY 60-6583 or netrin-1) could provide protection during ARDS by “drying out” the lungs (Eckle et al., 2008c; Eckle et al., 2013b; Hoegl et al., 2015; Wang et al., 2021b). In summary, these studies provide evidence from genetic and pharmacologic studies that netrin-1 is protective during ARDS, and implicate Adora2b signaling in mediating the observed lung protection (Figure 4).

### Inflammatory bowel disease

Inflammatory bowel disease (IBD) includes Crohn’s disease and ulcerative colitis, and is marked by persistent infiltration of the intestinal tissues with inflammatory cells. Due to its role orchestrating leukocyte trafficking, several studies have investigated the function of netrin-1 in IBD (Aherne et al., 2011; Aherne et al., 2013). Mice with partial netrin-1 deficiency (*Ntn1* ^*+/−*^) experience more profound weigh-loss and intestinal inflammation when exposed to dextran sulfate sodium (DSS) (Aherne et al., 2012). Since previous studies had shown a protective role of Adora2b signaling during DSS colitis (Eltzschig et al., 2009b; Frick et al., 2009; Aherne et al., 2015; Aherne et al., 2018) or intestinal ischemia and reperfusion (Hart et al., 2009), subsequent studies addressed the functional role of the Adora2b in netrin-1-elicited gut protection. For this purpose, the authors used an osmotic pump system to treat mice with recombinant netrin-1 during DSS colitis (Aherne et al., 2012). These studies demonstrated that wild-type mice that were treated with exogenous mouse netrin-1 experienced dramatically reduced intestinal inflammation, disease severity and weight loss. When those studies were repeated using gene-targeted mice deficient of the Adora2b, the treatment effects of recombinant netrin-1 delivered by osmotic pump were completely abolished (Aherne et al., 2012). Importantly, previous studies had shown that the Adora2b is induced by HIF1A, and implicate Adora2b signaling in attenuating inflammation in a variety of models of intestinal inflammation (Eltzschig et al., 2009b; Frick et al., 2009; Hart et al., 2009; Aherne et al., 2015; Aherne et al., 2018). Together, these studies implicate the netrin-1-Adora2b link in attenuating intestinal inflammation, as shown during inflammatory bowel disease.

### Myocardial infarction

**FIGURE 5:**
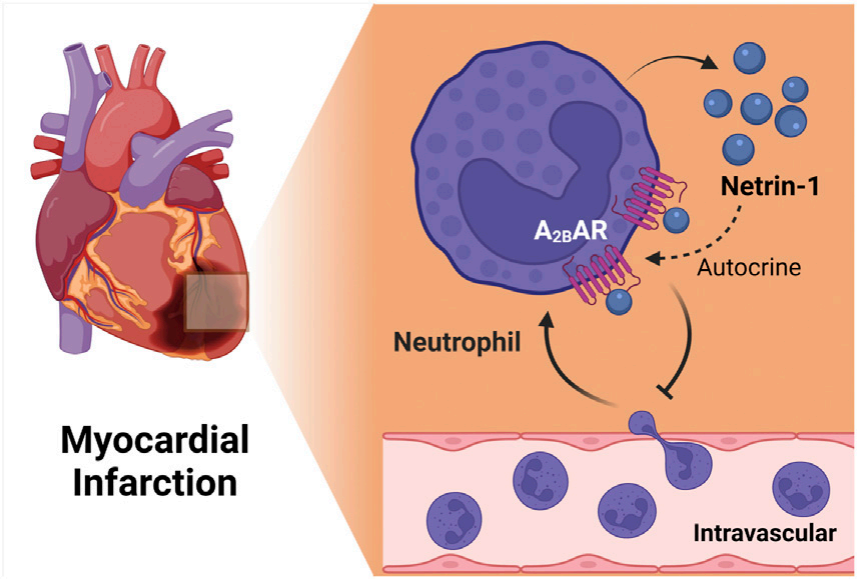
The netrin-1/Adora2b link in ischemia reperfusion injury of the heart. Myeloid cell derived netrin-1 has shown to be important for cardiac protection during ischemia reperfusion injury. Netrin-1 level increases during *in situ* myocardial infarction in mice and antibody mediated depetion of neutrophil abolished the induction, suggesting the importance of neutrophil as key sources of netrin-1. Furthermore, studies using treatment of recombinant netrin-1 implicated myeloid-dependent Adora2b signaling in cardioprotection, since the protection provided by netrin-1 treatment was completely abolished in *Ntrn1* ^*loxp/loxp*^ Myosin Cre + mice. Together, these findings implicate neutrophil-dependent netrin-1 release in mediating cardioprotection from ischemia and reperfusion by activating myeloid-dependent Adora2b adenosine receptors.

Myocardial ischemia and reperfusion injury is a leading cause of morbidity and mortality world-wide. Therefore, the search for novel therapeutic approaches to enhance myocardial resistance to ischemia or dampen myocardial reperfusion injury are areas of intense research (Eltzschig and Eckle, 2011; Heusch, 2020). Several previous studies have implicated netrin-1 signaling in attenuating myocardial ischemia and reperfusion injury (Mao et al., 2014), and have also identified signaling events related to classic netrin-1 receptors, e.g., *via* DCC signaling (Zhang and Cai, 2010; Bouhidel et al., 2015; Li et al., 2015). A recent study examined tissue-specific functions as well as the role of the netrin-1-Adora2b link (Li et al., 2021). This study showed increased circulating netrin-1 levels in patients suffering from myocardial infarction or in mice exposed to *in situ* myocardial ischemia and reperfusion injury. Tissue-specific studies suggested a myeloid source of netrin, since mice with myeloid netrin-1 deletion (*Ntrn1* ^*loxp/loxp*^ LysMCre+) experienced larger myocardial infarct sizes, and showed attenuated netrin-1 blood levels (Li et al., 2021). Interestingly, mice with myocardial netrin-1 deletion (*Ntrn1* ^*loxp/loxp*^ Myosin Cre + mice) had no phenotype with regard to myocardial injury. Subsequent studies using antibody mediated depletion (Lys6G) of PMNs (Neudecker et al., 2017b) implicated neutrophils as a key source for the cellular release of netrin-1 into the blood during myocardial injury (Li et al., 2021). After establishing a pharmacologic protocol to use recombinant netrin-1 for the treatment of myocardial injury, the authors deleted *Adora2b* from different tissue compartments in the heart (Eckle et al., 2012; Eltzschig et al., 2013; Seo et al., 2015). These studies directly implicated myeloid-dependent Adora2b signaling in cardioprotection, since the protection provided by netrin-1 treatment was abolished in *Ntrn1* ^*loxp/loxp*^ Myosin Cre + mice. Together, these findings implicate neutrophil-dependent netrin-1 release in mediating cardioprotection from ischemia and reperfusion by activating myeloid-dependent Adora2b adenosine receptors (Figure 5). These findings are also in line with previous studies showing a functional role of HIF in promoting Adora2b signaling during ischemia and reperfusion injury of the heart and cardioprotection (Eckle et al., 2008b; Eckle et al., 2012; Eltzschig et al., 2013; Koeppen et al., 2018; Lee et al., 2020b).

### Hepatic ischemia and reperfusion

Hepatic ischemia and reperfusion injury occurs during major liver surgery, or during liver transplantation, and represents a major hurdle towards improving outcomes in these clinical scenarios (Ju et al., 2016; Lee et al., 2020a; Cata et al., 2020; Conrad and Eltzschig, 2020). Previous studies had implicated hypoxia-signaling in liver protection, and provided a strong rationale for exploring the netrin-Adora2b link during hepatic ischemia and reperfusion injury (Eltzschig et al., 2009b; Gao et al., 2020; Ibars et al., 2020; Wang et al., 2021a; Ju et al., 2021; Kim et al., 2021). In this context it is not surprising that studies in mice with partial netrin-1 deletion (*Ntn1* ^*+/−*^) experienced lower efficacy in reducing neutrophil infiltration, had lower levels of pro-inflammatory cytokines, and exhibited attenuated liver injury during hepatic ischemia/reperfusion injury compared to wildtype control animals (Schlegel et al., 2016). Similarly, treatment with recombinant netrin-1 promoted liver protection and repair, attenuated neutrophil influx, and dampened liver injury, and also stimulated the endogenous biosynthesis of pro-resolving mediators and growth factors. Since these liver-protective signaling effects were abolished in *Adora2b* ^*−/−*^ mice, these studies directly implicate the netrin-Adora2b link in liver protection from ischemia and reperfusion injury (Schlegel et al., 2016).

### Other examples for the netrin-Adora2b link during inflammatory diseases

Several other studies of disease that occur at the interface between inflammation and hypoxia have provided additional evidence for the netrin-Adora2b link in the resolution of injury. For example, a recent study implicates netrin-1 in diabetic corneal wound healing through Adora2b signaling events (Zhang et al., 2018). Other studies demonstrate resolution of inflammatory peritonitis by activation of the Adora2b (Mirakaj et al., 2011). Again, other studies suggest a functional role of netrin-1 signaling during acute or chronic kidney injury and implicate the netrin-Adora2b link in promoting the resolution of injury (Grenz et al., 2011b; Tak et al., 2013). Finally, some studies have implicated netrin-1 signaling *via* the Adora2b in the treatment of *Aspergillus fumigatus* infection of the cornea (Zhou et al., 2019). Taken together, these studies during inflammation or ischemia and reperfusion provide evidence for the netrin-Adora2b link in attenuating inflammation, promoting the resolution of inflammation and rescuing organ function (Aherne et al., 2013; Mirakaj and Rosenberger, 2017; Keller et al., 2021).

## Does netrin-1 function as a direct agonist of the Adora2b adenosine receptor?

The original report that identified the netrin-Adora2b link used a two-hybrid screen with the intracellular DCC domain as a bait, and identified binding of DCC and Adora2b intracellular domains (Corset et al., 2000). Subsequently, these studies indicated that netrin-1 signaling through the Adora2b promotes cAMP levels, and suggest the Adora2b as a direct netrin-1 receptor (Corset et al., 2000). Although intriguing, the issue of a direct interaction between netrin-1 and the Adora2b is controversial. For example, a subsequent study provides evidence that netrin-1-independent of Adora2b signaling-controls the responsiveness of neurons to netrin-1 by alternating cell surface UNC5A receptors (McKenna et al., 2008). As part of those studies, the authors demonstrate that COS cells with overexpression of the Adora2b did not show binding to this receptor, or responded with intracellular signal transduction when stimulated by netrin-1 (McKenna et al., 2008). On the other hand, *in vitro* studies of PMN transmigration following a chemotactic gradient was shown to be effectively attenuated in the presence of netrin-1, a signaling effect of netrin-1 on PMNs that could be effectively inhibited in the presence of a specific Adora2b agonist (PSB1115), or by using PMNs from *Adora2b* ^*−/−*^ mice, implicating a direct functional role of Adora2b signaling in netrin-1-mediated inhibition of inflammatory responses (Rosenberger et al., 2009).

**FIGURE 6:**
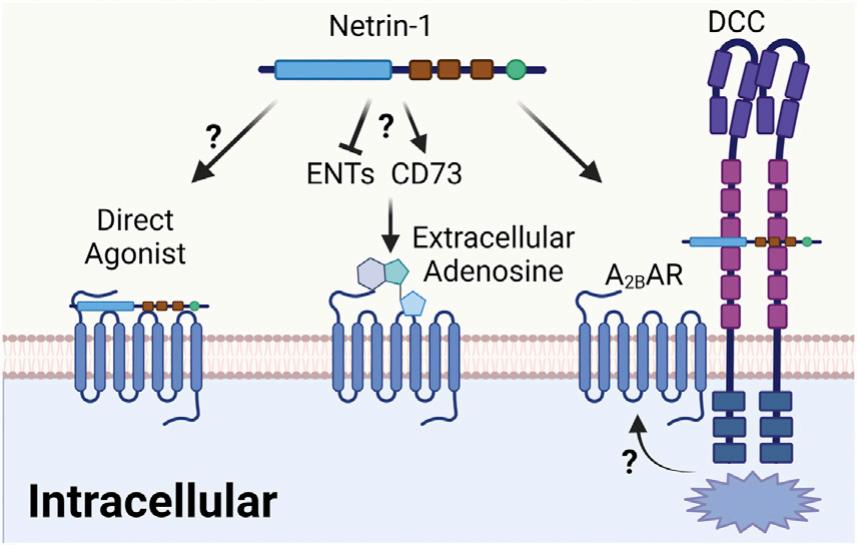
Proposed mechanism of the netrin-1/Adora2b linkage. The interaction between netrin-1 and Adora2b could have several different mechanisms. Firstly, the issue of a direct interaction between netrin-1 and the Adora2b is still controversial, although previous study has indicated netrin-1 as direct agonist. Alternatively, netrin-1 could function to enhance extracellular adenosine levels, and thereby promote anti-inflammatory signaling pathways that are under the control of the Adora2b. Such mechanisms could potentially involve increases in extracellular adenosine generation by activation of CD73 or inhibition of extracellular adenosine update *via* ENTs. An additional alternative of how netrin-1 signaling could enhance Adora2b signaling could involve an interaction of netrin-1 with a classic netrin-1 receptor, such as the DCC, which was indicated in previous studies. This could argue for a signaling pathway where netrin-1 binds to DCC and an interaction between DCC and the Adora2b promotes intracellular signaling cascades that are in line with Adora2b signaling.

In addition to conflicting findings regarding the potential activity of netrin-1 on the Adora2b, it also remains unclear how these signaling mechanisms occur from a molecular perspective. While the original description of the netrin-Adora2b link postulates a direct effect of netrin-1 as an endogenous Adora2b agonist (Corset et al., 2000), there are other models that could explain how netrin-1 would enhance Adora2b signaling without functioning as a direct Adora2b agonist (Figure 6). First, it is conceivable that netrin-1 functions to enhance extracellular adenosine levels, and thereby promote anti-inflammatory signaling pathways that are under the control of the Adora2b. Such mechanisms could potentially involve increases in extracellular adenosine generation by activation of CD73. Alternatively, netrin-1 could function to inhibit extracellular adenosine update *via* ENTs or intracellular adenosine metabolism by inhibiting adenosine kinase or adenosine deaminase (Eltzschig et al., 2006c; Van Linden and Eltzschig, 2007; Morote-Garcia et al., 2008). A recent study argues against this theory. In this study, the authors found that the presence of myeloid Adora2b receptors is necessary to mediate the cardioprotective effects of treatment with recombinant netrin-1 (Li et al., 2021). However, measurements of cardiac or circulating levels of adenosine were not altered by treatment doses of recombinant netrin-1 that were associated with attenuated myocardial infarct sizes (Li et al., 2021). An additional alternative explanation for how netrin-1 signaling could enhance Adora2b signaling involves a potential interaction of netrin-1 with a classic netrin-1 receptor, such as the DCC. In fact, the first description of Adora2b and netrin-1 signaling demonstrates an association of the Adora2b with DCC and netrin-1 by co-immunoprecipitation (Corset et al., 2000). This could argue for a signaling pathway where netrin-1 binds to DCC and an interaction between DCC and the Adora2b promotes intracellular signaling cascades that are in line with Adora2b signaling (Figure 6). Further molecular studies would be required to characterize the molecular events that govern netrin-1-elicited enhancements of Adora2b signaling.

## Summary and discussion

Many studies support the notion that extracellular adenosine signaling is enhanced during limited oxygen availability, such as occur during ischemia or inflammatory diseases (Colgan et al., 2006; Eltzschig et al., 2008; Colgan and Eltzschig, 2012; Eltzschig et al., 2014). Signaling events through Adora2b have been shown to dampen inflammatory hypoxia during organ injury (Bowser et al., 2017b; Sun et al., 2017; Yuan et al., 2017; Bowser et al., 2018; Le et al., 2019; Li et al., 2020). Several studies have implicated netrin-1 in utilizing this pathway as a means of alternative activation of Adora2b signaling. While many of these studies implicate netrin-1 in Adora2b signaling, the detailed molecular mechanisms of netrin-1-dependent Adora2b signaling have yet to be further characterized from a molecular perspective. In addition, clinical studies using this pathway would be desirable for the treatment of inflammatory or ischemic diseases. There could be several advantages of treatments with netrin-1 over other clinical strategies to enhance extracellular adenosine signaling through the Adora2b. First, netrin-1 has a much longer half-life than extracellular adenosine signaling, which has always been a concern about the use of direct adenosine treatment strategies (e.g. intravenous adenosine infusions). Secondly, unwanted side-effects of adenosine treatments (e.g. bradycardia or hypotension) may be less pronounced when using recombinant netrin. While it is unclear why direct Adora2b agonists have not been examined in clinical trials (e.g. BAY 60-6583) (Chen et al., 2009; Hart et al., 2009; Koscso et al., 2013), treatment with recombinant netrin-1 may be beneficial since netrin-1 represents an endogenous anti-inflammatory compound, and could therefore be safer and better tolerated as compared to an “engineered” pharmacologic Adora2b agonist.
